# Orchestration of autonomous trusted third-party banking

**DOI:** 10.12688/f1000research.72987.1

**Published:** 2021-09-08

**Authors:** Saravanan Muthaiyah, Kalaiarasi Sonai Muthu Anbananthen, Nguyen Thi Phuong Lan

**Affiliations:** 1 Faculty of Management, Multimedia University, Cyberjaya, Selangor, 40400, Malaysia; 2 Faculty of Information Science and Technology, Multimedia University, Bukit Beruang, Melaka, 75450, Malaysia

**Keywords:** Autonomous banking, RkBAC, RBAC, task delegation, risk ordering relation, role based, risk band, risk graph

## Abstract

*Background*

Digital transformation is changing the structure and landscape of future banking needs with much emphasis on value creation. Autonomous banking solutions must incorporate on-the-fly processing for risky transactions to create this value. In an autonomous environment, access control with role and trust delegation has been said to be highly relevant. The aim of this research is to provide an end to end working solution that will enable autonomous transaction and task processing for banking.

*Method*

We illustrate the use case for task delegation with the aid of risk graphs, risk bands and finite state machines. This paper also highlights a step by step task delegation process using a risk ordering relation methodology that can be embedded into smart contracts.

*Results*

Task delegation with risk ordering relation is illustrated with six process owners that share immutable ledgers. Task delegation properties using Multi Agent Systems (MAS) is used to eliminate barriers for autonomous transaction processing. Secondly, the application of risk graph and risk ordering relation with reference to delegation of tasks is a novel approach that is nonexistent in RBAC.

*Conclusion*

The novelty of this study is the logic for task delegation and task policies for autonomous execution on autonomous banking platforms akin to the idea of federated ID (Liberty Alliance).

## Introduction

**Figure 1.**
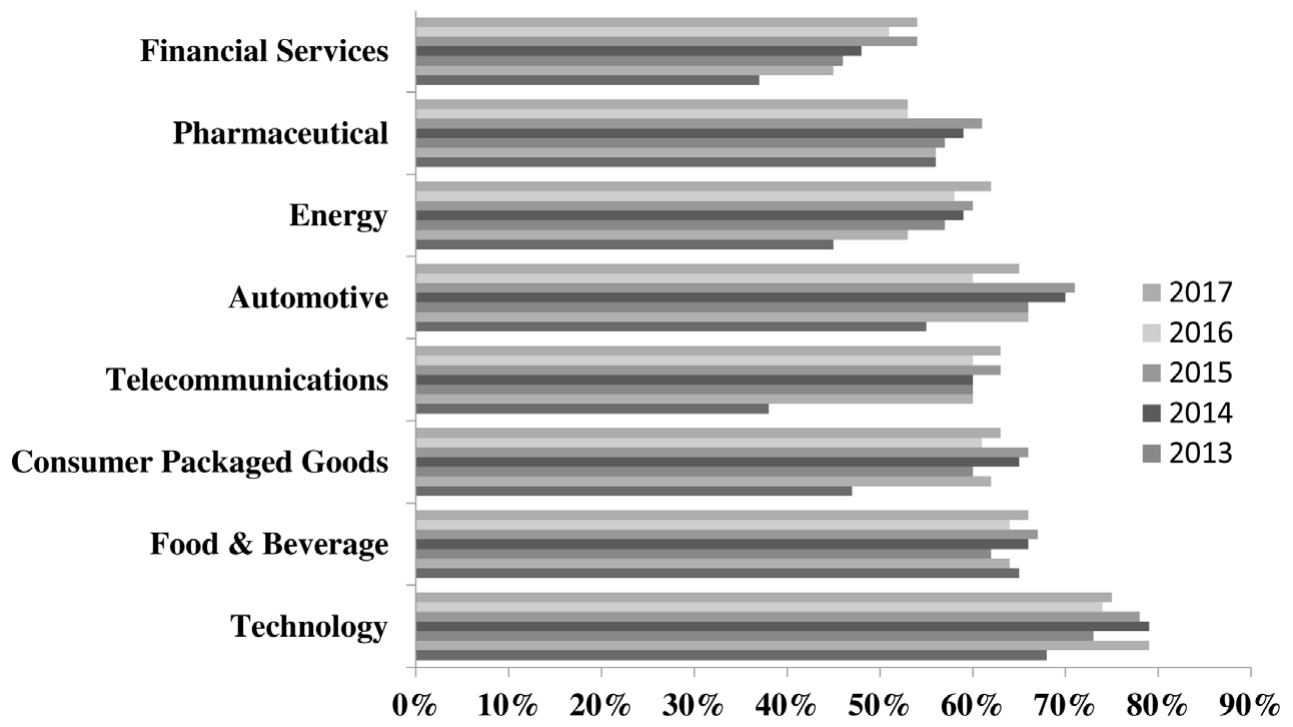
Edelman and Trust Report on Trusted Industries (2011 to 2017).

Digital transformation has been changing the landscape of banking and the future of banking will be very much different from what it is today. Faced with enormous competition, consumer expectations and new business models’ banks are required to put in place process automation that will gain confidence of its customers. With much negative publicity from recent events such as Enron, Madoff Investment Securities and WorldCom, the financial sector is becoming the least trusted sector. This is constantly highlighted in the Edelman Trust Barometer report. From 2011 until 2017, among eight industries financial services that incudes baking has been reported as the least trusted. In 2011 the sector scored 37% and in 2017 it scored 54%. The percentage increase is negligible compared to the other industries as shown in Figure 1. Financial services must embrace trust in its core business model in order to overcome this negative perception. Future of banking will include autonomous systems that must ensure trust at it’s core processing. In this paper we present a Multi Agent System (MAS) approach that is based on the Blockchain technology to facilitate data exchange, loan processing, withdrawals, loan inquiry, third party transfers, wallet activation and much more.

This is to ensure customer confidence and trust is ensured ^1^. The core of this technology ensures provenance, data integrity, auditability and trust ^2^. The decentralized trust embedded system has been illustrated with the Trusted Third Party (TTP) principle ^3^.

## Trust attributes

**Figure 2.**
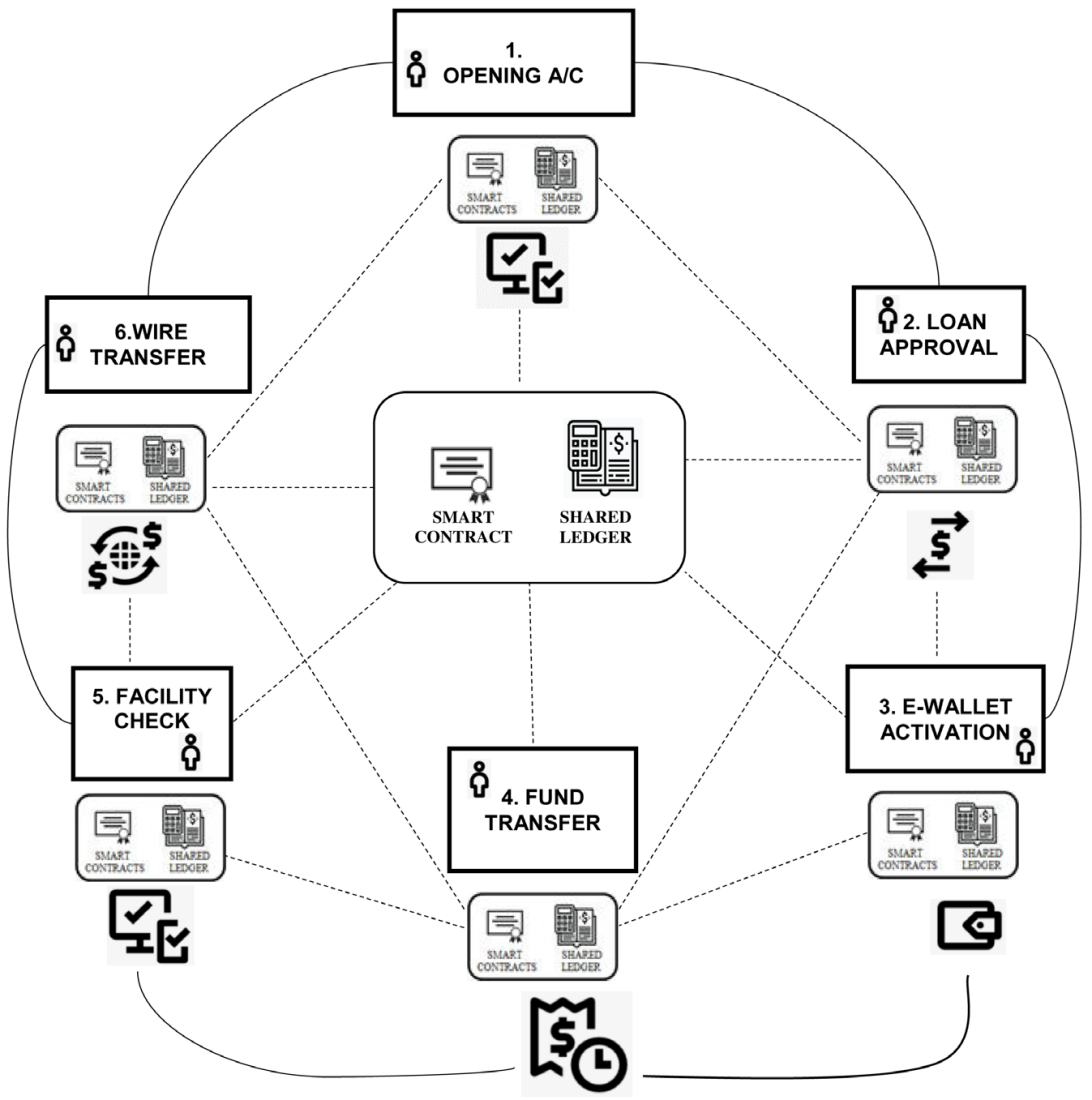
Shared Immutable Ledger (Muthaiyah, 2019).

Trusted systems are defined as systems that rely upon upholding or enforcing trust in relation to transaction processing, integrity, data provenance, auditability and adherence to policy. In an autonomous banking system trust attributes can also be defined as compliance, data provenance as well as true and fairness. The purpose of statutory audit is to ensure compliance and rigor for check and balance. Blockchain technology is useful in this context to enable a unified vision that is agreeable and verifiable by all entities involved in the trusted network ^3, 4^. As mentioned earlier to facilitate Tasks (t1, t2…,.tn) that refer to specific task descriptions highlighted in Table 1, we propose evaluation of Exposure Analysis (EA) and Risk Band (RB) ^5^. The idea is to specifically incorporate trust factor into the distributed ledgers via smart contracts that will provide required governance for all transactions illustrated in Figure 2 below.

**Table 1.** Automated Risk Band (RB) Output.

Tasks (T)	RR (Risk Rating) %	EA (Exposure Analysis)	EL (Expected Loss)	RB (Risk Band)
T1	79%	USD $ 6,300	USD$ 4977	High risk
T2	70%	USD $ 5,500	USD$ 3850	High risk
T3	69%	USD $ 5,800	USD$ 4002	Medium risk
T4	59%	USD $ 7,000	USD$ 4130	High risk
T5	57%	USD $ 6,800	USD$ 3876	Medium risk
T6	50%	USD $ 5,900	USD$ 2950	Medium risk
T7	42%	USD $ 4,500	USD$ 1890	Low risk
T8	39%	USD $ 4,300	USD$ 1677	Low risk
T9	35%	USD $ 3,900	USD$ 1365	Low risk
T10	33%	USD $ 3,500	USD$ 1155	Low risk

In this instance, we highlight a transaction that is shared across six process owners. The immutable ledgers will evaluate individually for each process owners i.e. 1) opening account, 2) loan approval, 3) e-wallet activation, 4) fund transfer, 5) facility check and 6) wire transfer.

## Methods

In this study, the RR, EA and EL analytical data model below was used to rank order to determine Risk Band (RB) based on ISO31000 standards. Multi Agent Systems (MAS) will execute and update RB into respective immutable ledgers shown in Figure 2.

### Statecharts

**Figure 3.**
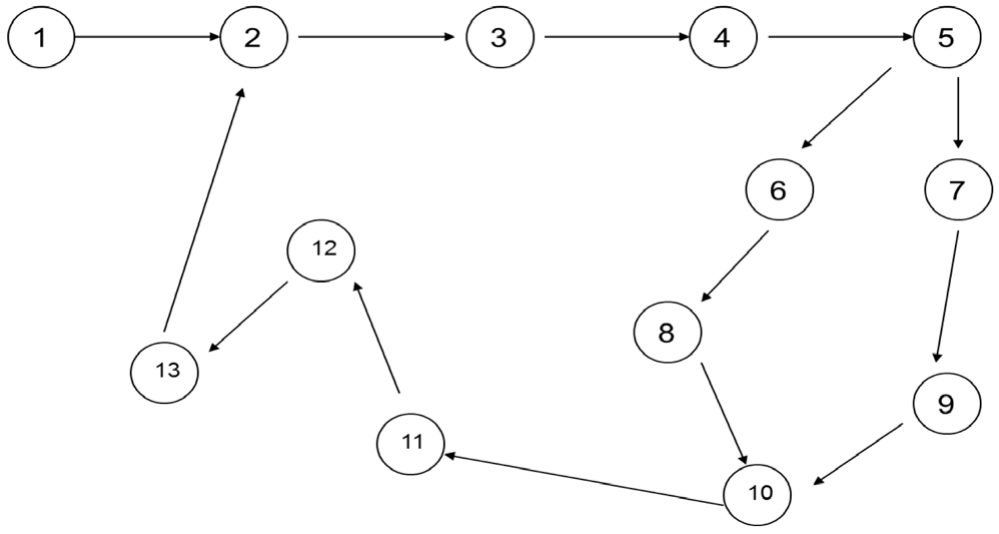
State chart diagram.

State charts or state chart diagrams can be used to define processes that are dynamic. It is used to define state changes that are triggered by events ^6^. As depicted in Figure 3, there are thirteen states that are being triggered one after the other. Task 1 (t1) triggers task 2 (t2) until all thirteen states are completed for a particular transaction. As mentioned earlier, these tasks are transaction based such as a third-party fund transfer. Since the banking system is designed to be autonomous the MAS will coordinate the automated transaction processing without human intervention.

**Figure 4.**
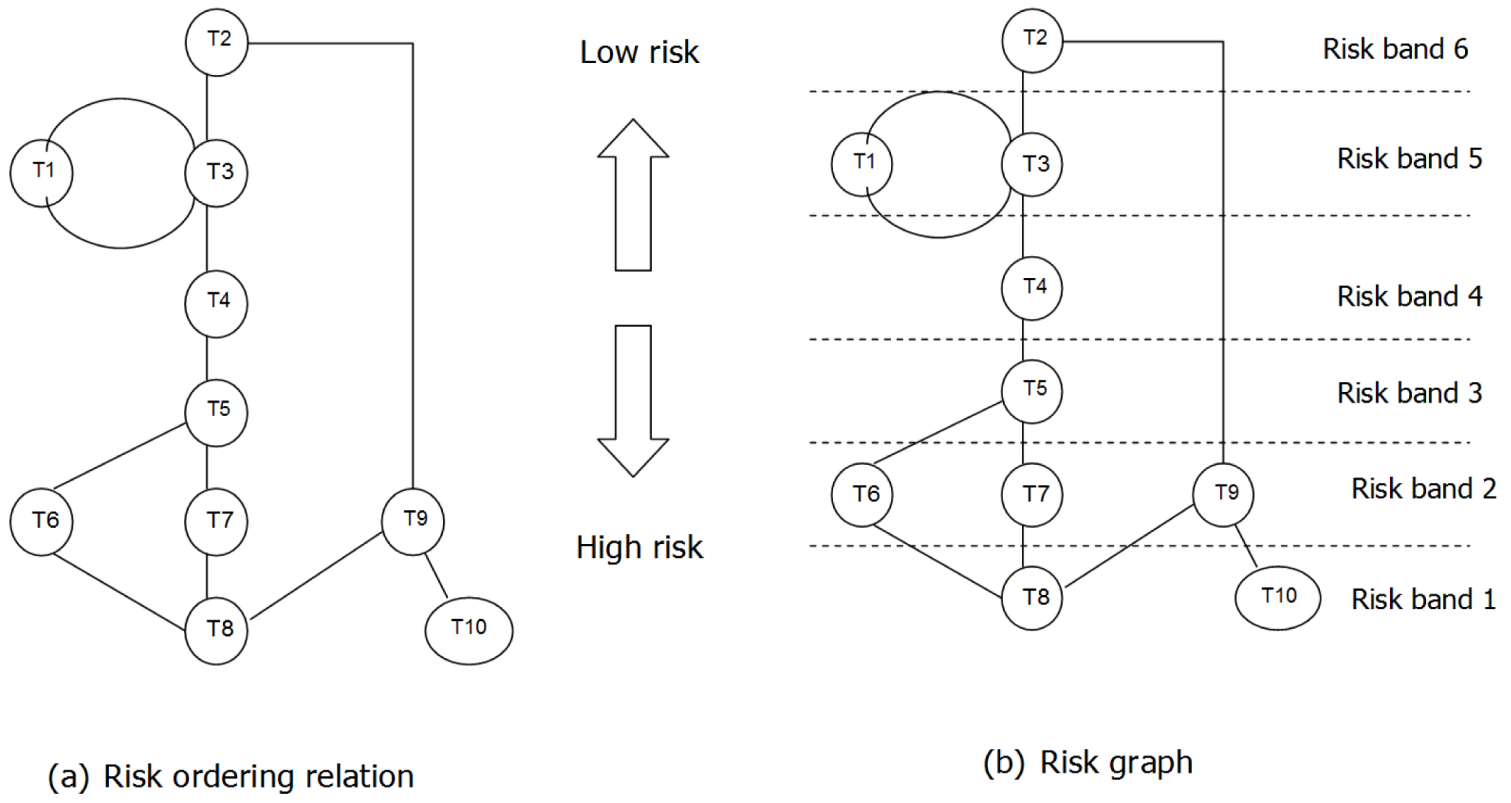
Risk Graph associated with Risk Band (RB).

Table 2 illustrates nine tasks with relation to banking transactions without trust delegation. Description of the tasks (T) and relative Risk Bands (RB) have also been listed. RB can be grouped into five groups of risks, 1) Low risk, 2) Low to Medium, 3) Medium risk, 4) Medium to High risk and 5) High risk. Risks are identified by RB and the risk levels are indicated numerically from 1 to 6 as shown in Figure 4. Low level of risk is indicated by RB5 and RB6 that maps to T2 (statement request) and T1 (opening account). High level of risk is indicated by RB1 that maps to T8 as well as T10. In the Figure 1 below, there are nine KYPs (t1…t9), which are tagged to company platforms available on the P2P platform.

**Table 2.** Automated Transaction.

Tasks (T)	Description / Process	Risk Band (RB)
T1	Opening account	Low risk
T2	Statement request	Low risk
T3	Personal loan approval	Low to medium risk
T4	Cheque status inquiry	Medium risk
T5	E-Wallet activation	Medium to high risk
T6	Stop cheque request	Medium to high risk
T7	Fund transfer	Medium to high risk
T8	Loan facility approval	High risk
T9	Loan facility increase	High risk
T10	International foreign currency wire transfer	High risk

Analysis from RA and EA calculations show that Risk band 6 (RB = 6) includes t1, t2, and t4, which are high risk platforms. Risk band 1 (RB = 1) includes t7, t8 and t9, which are relatively low risk platforms. Overall, we can compare the risk bands to show relative risks between (t1 to t9). For example, t9, will have the same level of risk as t8, which are compatible in terms of risk band. T8 refers to Loan Facility Approval and T10 refers to international foreign currency wire transfer. Comparable risks are for tasks (T6, T7 and T9) which all fall under RB2. Similarly, T1 and T3 also share the same RB which is RB5. Whereas non-comparable RBs are those such as different tiers such as T9 and T10.

### Trust attributes and risk band

Risk scales for automated transaction are embedded into our proposed autonomous banking system for enabling a trust-based network. The main objective of this study is to determine access rights based on risk bands. The idea is to use risk-based assessment for better control measure and execution. Intuitively, the larger the risk, the greater the risk band and the higher the scrutiny. Before rights to access is granted, detailed access control permissions are allocated based on risk bands. However, trust delegation for transactions can also be dealt with in this methodology. This sis explained further in the next section.

### Trust delegation

Trust delegation in principle refers to any task (t) where the role of approval can be delegated or transferred personnel within boundaries of process owners ^7^.

**Figure 5.**
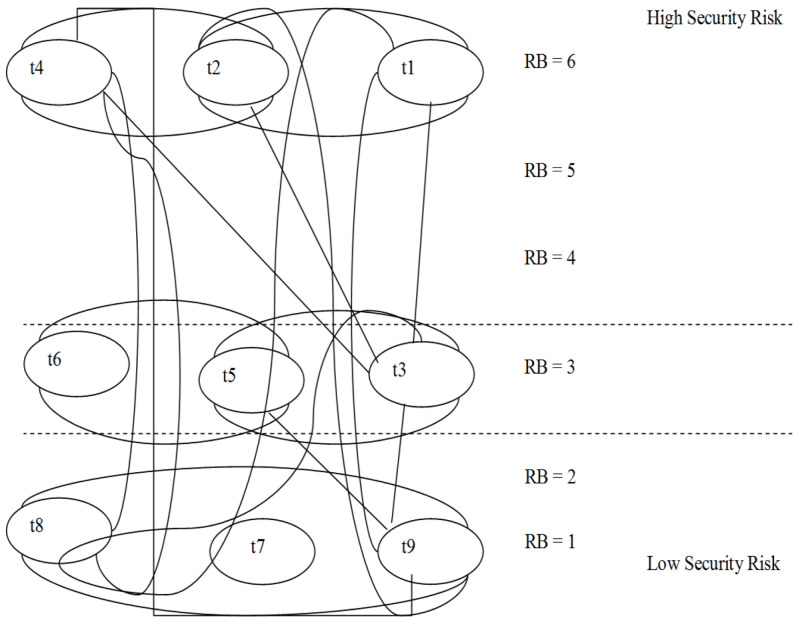
Risk Graph with Trust Delegation (Muthaiyah, 2020).

Figure 5 highlights the proposed trust based autonomous banking platform where trust delegation is included. A total of nine tasks (t1…t9), for autonomous banking is illustrated. Risk band 6 (RB6) includes high risk tasks (t1, t2 and t4). (RB1) includes low risk tasks (t7, t8 and t9). Risk band 3 (RB3) includes average risk tasks (t3, t5 and t6).

For example, if a process owner executes t9, the process owner will have the same level of risk as t8, which are compatible in terms of risk band. Any task that has a higher risk than RB = 1, more scrutiny will be applied to grant permission for that task.

This flexibility is crucial for tasks that are dynamic in the context of autonomous banking. In this manner post evaluation for the transactions can be executed this enabling risk levels to move up or down depending on the RB. Intuitively, the larger the gap between the RB, the higher the risk ^7^. As such, Tasks t1, t2, and t4 belong to the same level of risk (RB = 6). Securing transaction threats by accessing risks associated with them can reduce likelihood of liabilities however the risk assessment process should not be ambiguous, inconsistent and have omissions. Therefore, the mathematical formulation below is necessary to add rigor to the assessment ^7^.

**Figure 6.**
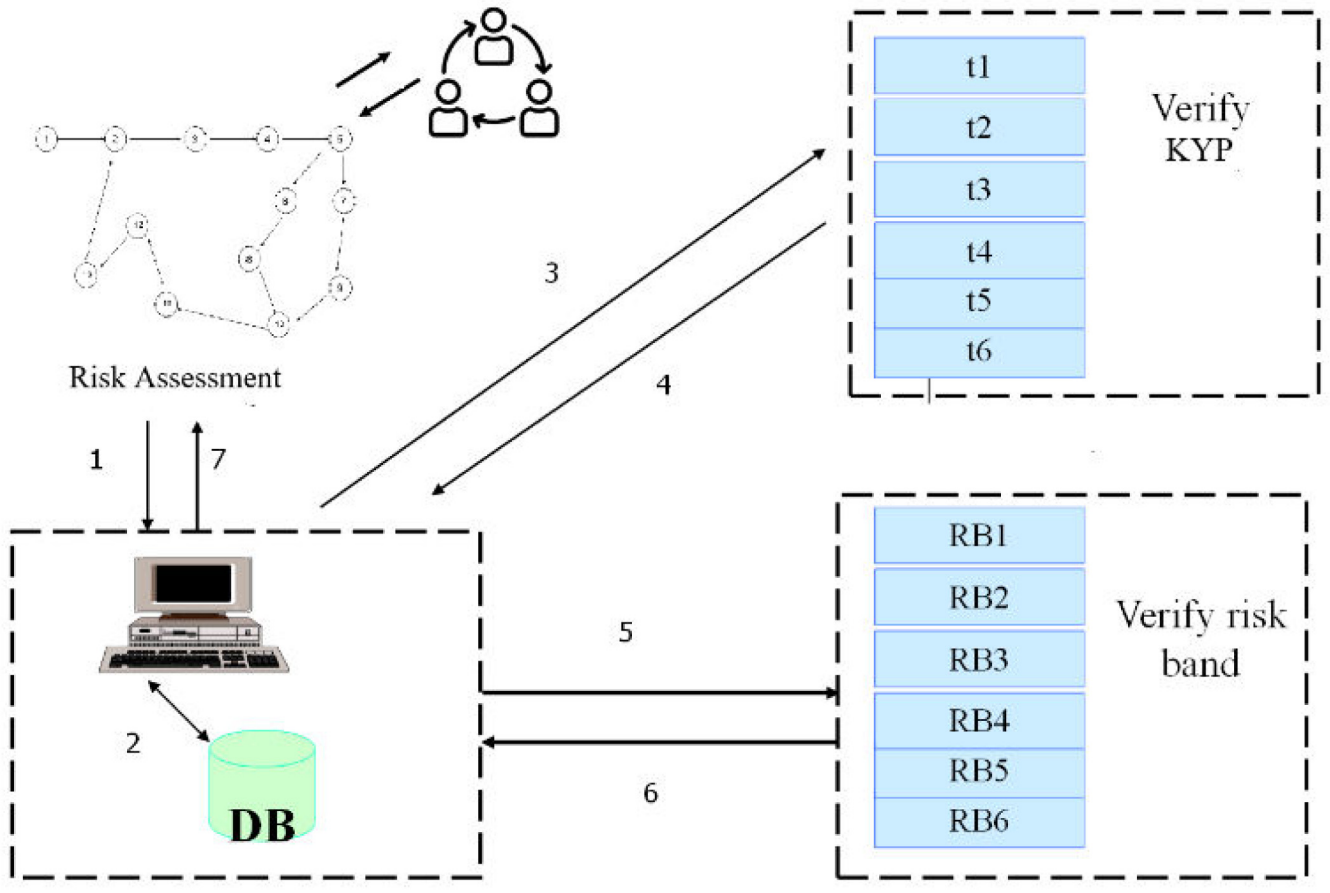
Agent Systems for autonomous platforms (Muthaiyah, 2020).

In a Multi Agent System (MAS) platform for autonomous systems in this case “agents”, there is a need to have the agent systems programmed with a certain logic or algorithm so that they can execute these transactions seamlessly. As such we illustrate the following mathematical expression for developing the logic for risk ordering and risk bands. We have implemented a detailed algorithm using the Foundation of Intelligent Physical Agents (FIPA) standard shown in Figure 6.

1) Subject (S) – all possible users as well as non-human entities (i.e. FSMs)
2) Object (O) – entities being accessed by subject
3) Operation (Op) – operation performed on object (i.e. loan processing)
4) Role (R) – Capacity in which subjects access the rights to objects
5) Task (T) – Operation * Object (Op * O)
6) Permission (P) – Role (R) → P Task (T) (Note : P denotes power set operand)
  - gives a set of tasks authorized for each role (R).
  - task has a token and when task is over the token would expire.
7) Subject Roles (SR) – Subject (S) → P Role
8) PERM is a subset of Role * Task (R*T)

However, this paper’s focus is only on developing the logic for trust delegation at this stage. More detailed transaction logic will be implemented it in the near future to accommodate transactions that are not listed in Table 2. Figure 6, illustrates autonomous banking trust delegation for task execution workflow ^5^. The concept is based on a Multi Agent Systems (MAS) platform assumed by the functional architecture proposed by FIPA, an Agents Working Group. MAS can be thought of multitude autonomous entities, that execute processes one through seven shown below. This paper presents an implementation of an autonomous banking platform providing transparent interaction between process owners that are represented by agent systems.

## Conclusion

Autonomous Trusted Third-Party orchestration for banking systems must be self-auditing by its design. In order to ensure that the banking ecosystem will entail trust which will be the key to drive of its success. Technological advancements will be able to create value by quickly aggregating data which can be deployed using agent systems. In our future work we would like to investigate how control procedures can be embedded within the Blockchain technology to make autonomous banking platforms more robust.

## Data availability

No data associated with this article.

### Ethics

Ethical Approval Number: EA1202021

Ethical approval was obtained from the research management center at the university. Researchers had to first submit the title of the project, what the author planned to do for the interviews and details of study objectives. The officer at the research management center after reviewing the documents will then issue a letter of clearance for the data collection to be carried out. The approval letter was then obtained, and the reference number of this letter is EA1202021.
