# Supplementary material for: Orchestration of autonomous trusted third-party banking
Source: F1000Res. 2021 Sep 8;10:899. [Version 1] doi: 10.12688/f1000research.72987.1 (PMC8543169; doi:10.12688/f1000research.72987.1)
Supplement: Peer review history

# Peer review: Reviewer response for version 1

**DOI:** 10.5256/f1000research.76604.r96360

**Published:** 2021-11-16

**Authors:** Kadambini Katke

**Affiliations:** 1 Dayananda Sagar Institutions, Bangalore, Karnataka, India

1. Industry relevant research area which can contribute towards digitization of banking services 

 2. Paper can be improved with a few more added references to support the research findings

 3. Self-citations out of a total of seven references results in limited literature coverage. 

 4. Research findings and flow charts are satisfactory and supports the title

Is the work clearly and accurately presented and does it cite the current literature?

Yes

If applicable, is the statistical analysis and its interpretation appropriate?

Yes

Are all the source data underlying the results available to ensure full reproducibility?

Yes

Is the study design appropriate and is the work technically sound?

Yes

Are the conclusions drawn adequately supported by the results?

Yes

Are sufficient details of methods and analysis provided to allow replication by others?

Yes

Reviewer Expertise:

technology management, general management, marketing management.

I confirm that I have read this submission and believe that I have an appropriate level of expertise to confirm that it is of an acceptable scientific standard, however I have significant reservations, as outlined above.

# Peer review: Reviewer response for version 1

**DOI:** 10.5256/f1000research.76604.r96362

**Published:** 2021-10-22

**Authors:** Noor Ismawati Jaafar

**Affiliations:** 1 Department of Operations and MIS, Faculty of Business and Accountancy, University of Malaya, Kuala Lumpur, Malaysia

In general, this may be a good start to more interesting research and findings. The context and focus of the research are very interesting and have huge potential to make an impact on society's application of IT, especially in the banking environment. Although I am not very familiar with the technique used in the article, this research has its own merit and contribution to the fintech environment and the banking industry as a whole. The use of blockchain and smart ledgers is an interesting phenomenon to study. What is good about this article is the assigning of risk band in terms of the 6 common processes in the banking environment. The use of RR, EA, EL, and MAS as stated by the author makes this article worth reading and understanding. These methods used as an approach in developing the risk band enhance the strength of the article. Moreover, the issue of trust that becomes the focal point in the article has been given the attention that links between the customers and the bank.

 The results would definitely serve as reference points for the experts like IT auditors to assess or review the banking environment in the future. By defining the risk band and exposure analysis, this article has highlighted an interesting perspective on trust in the banking industry. I would also like to touch on the task delegation and policies for autonomous execution which could be adopted in the future banking environment. This could definitely benefit the banks in terms of efficiency and better services quality to the customers. Thus, the short simplistic article presented has served as a wake-up call for operations and IT researchers to expand the study on trust issues in the financial and banking environment.

Is the work clearly and accurately presented and does it cite the current literature?

Yes

If applicable, is the statistical analysis and its interpretation appropriate?

I cannot comment. A qualified statistician is required.

Are all the source data underlying the results available to ensure full reproducibility?

Yes

Is the study design appropriate and is the work technically sound?

Yes

Are the conclusions drawn adequately supported by the results?

Yes

Are sufficient details of methods and analysis provided to allow replication by others?

Yes

Reviewer Expertise:

It governance, AIS

I confirm that I have read this submission and believe that I have an appropriate level of expertise to confirm that it is of an acceptable scientific standard.

# Peer review: Reviewer response for version 1

**DOI:** 10.5256/f1000research.76604.r93739

**Published:** 2021-10-12

**Authors:** Indrawati Sambas

**Affiliations:** 1 Faculty Economics & Business, Telkom University, Bandung, Indonesia

I think this paper is a good and important addition to the literature. I really like the conceptualization of how autonomous Trusted Third-Party orchestration for banking systems must be self-auditing by its design which I think is very important in today's banking industry. This article addresses the links between the emerging of Blockchain technology and the need for a self-auditing process in the banking industry.  They explained the specific incorporation of the trust factor into the distributed ledgers via smart contracts in blockchains that enable the provision of required governance for all transactions. Ensure that the banking ecosystem will entail trust is the key to drive its success.

 Implementing the RR, EA, and EL analytical data model to determine Risk Band (RB) based on ISO31000 standards and Multi-Agent Systems (MAS) that executing and updating RB into respective immutable ledgers are excellent. This study explained how logic for task delegation and task policies for autonomous execution on autonomous banking platforms run.  I do hope that the future work they will investigate how control procedures can be embedded within Blockchain technology to make autonomous banking platforms more robust.

Is the work clearly and accurately presented and does it cite the current literature?

Yes

If applicable, is the statistical analysis and its interpretation appropriate?

Yes

Are all the source data underlying the results available to ensure full reproducibility?

Yes

Is the study design appropriate and is the work technically sound?

Yes

Are the conclusions drawn adequately supported by the results?

Yes

Are sufficient details of methods and analysis provided to allow replication by others?

Yes

Reviewer Expertise:

My area of research are technology adoption including block chain adoption in the area of finance and marketing.

I confirm that I have read this submission and believe that I have an appropriate level of expertise to confirm that it is of an acceptable scientific standard.
